# AAL and Internet of Medical Things for Monitoring Type-2 Diabetic Patients

**DOI:** 10.3390/diagnostics12112739

**Published:** 2022-11-09

**Authors:** Shakeel Ahmed, Parvathaneni Naga Srinivasu, Abdulaziz Alhumam, Mohammed Alarfaj

**Affiliations:** 1 Department of Computer Science, College of Computer Sciences and Information Technology, King Faisal University, Al-Ahsa 31982, Saudi Arabia; 2 Department of Computer Science and Engineering, Prasad V Potluri Siddhartha Institute of Technology, Vijayawada 520007, India; 3 Department of Electrical Engineering, College of Engineering, King Faisal University, Al-Ahsa 31982, Saudi Arabia

**Keywords:** AAL, IoMT, Type-2 diabetes, biosensors, DenseNet-169, hyperparameters

## Abstract

Due to an aging population, assisted-care options are required so that senior citizens may maintain their independence at home for a longer time and rely less on caretakers. Ambient Assisted Living (AAL) encourages the creation of solutions that can help to optimize the environment for senior citizens with assistance while greatly reducing their challenges. A framework based on the Internet of Medical Things (IoMT) is used in the current study for the implementation of AAL technology to help patients with Type-2 diabetes. A glucose oxide sensor is used to monitor diabetic elderly people continuously. Spectrogram images are created from the recorded data from the sensor to assess and detect aberrant glucose levels. DenseNet-169 examines and analyzes the spectrogram pictures, and messages are sent to caregivers when aberrant glucose levels are detected. The current work describes both the spectrogram image analysis and the signal-to-spectrogram generating method. The study presents a future perspective model for a mobile application for real-time patient monitoring. Benchmark metrics evaluate the application’s performances, including sensitivity, specificity, accuracy, and F1-score. Several cross--validations are used to evaluate the model’s performance. The findings demonstrate that the proposed model can correctly identify patients with abnormal blood glucose levels.

## 1. Introduction

**Figure 1:**
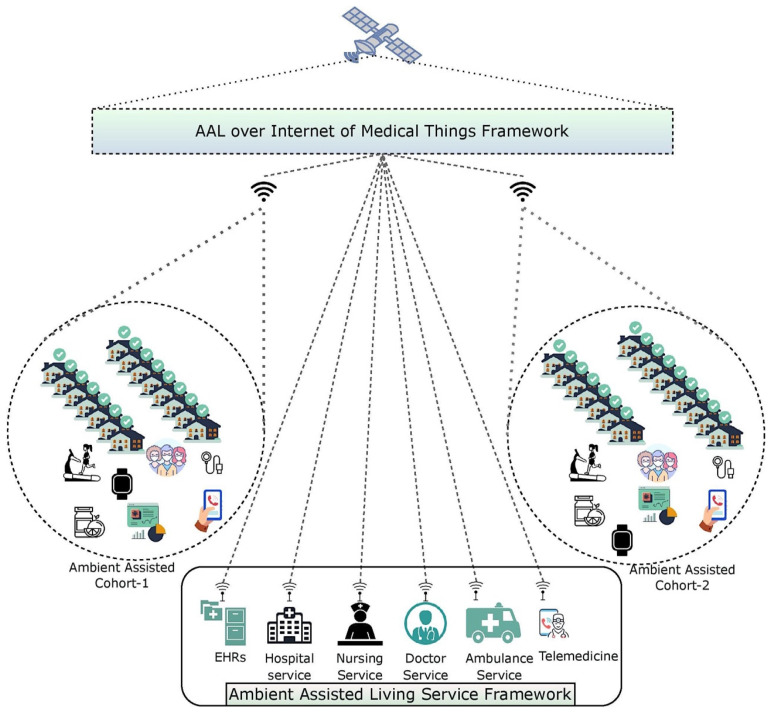
Image representing the AAL services over IoMT architecture.

In affluent nations, a rising number of older individuals wish or are required to live as autonomously as possible in their later years. Revolutionary technology such as Ambient Assisted-Living solutions can support and ease the daily lives of the elderly, extending the time they can live independently and supporting professional and volunteer caregivers. On the other hand, caregivers can be spared from unneeded and routine checks on the elderly, allowing them to use their time effectively and to be where their presence is needed to support their clients. AAL involves the use of ICT infrastructure in a person’s way of life to continue to be active longer, stay socially engaged, and lead an everyday life into old age [1]. AAL implements and uses clever technology to help the elderly remain longer in their preferred surroundings. There has been an explosion in the last decade in the number of technology solutions for the care of the elderly and the physically challenged. Personal coaching, activity monitoring, health-parameter tracking, emergency detection, and other features are all possible with AL systems. User mobility and motion are two of the essential indicators of a person’s well-being, and they may be monitored by various technologies and utilized as contextual information for other systems and products. Older adults’ daily routines and movement patterns are important in healthcare applications, since they may help identify changes in habits, odd actions, or a worsening state of health. Such information must be shared with caretakers, physicians, or other members of the user’s social network [2]. The architecture of the proposed AAL technology over the IoMT framework is presented in Figure 1.

Older persons who were concerned about the impact of COVID-19 on their life, according to recently published studies [3], were most concerned about their ability to make plans and to engage in activities that influence their well-being. Remote communication with loved ones was cited as a technique to combat the feeling of isolation. Various Ambient or active assisted-living approaches have been developed to help senior citizens live independently and safely in their environment, as technological advances, such as the integration of various cross-domain aspects, e.g., the internet of things (IoT), machine intelligence, sensors, cloud computing, cellular technologies, and assistive robotics, have emerged in recent decades. With these innovations, older adults can better preserve their physical and psychological well-being and improve the quality of their lives in their local communities [4].

To enhance data collection in AAL contexts, the Internet of Medical Things (IoMT), a development of the Internet of Things (IoT), has also been established. Many stakeholders may benefit from this information, and the AAL can recommend services to help them achieve their goals. When it comes down to it, IoMT’s main goal is to bring together people, data, progress, and mobile apps to monitor patient health outcomes. Remote patient monitoring is the most widely used IoMT-connected development for keeping tabs on a patient’s health. Healthcare providers’ networks transmit data from their devices to cloud servers. Communication security is the most important aspect of IoMT networks, since it protects patient privacy and safety. IoMT is undoubtedly the most acceptable solution to streamline clinical workflows and enhance patient outcomes. Accurate diagnoses and minimal expenses will be achieved using IoMT implementation. Machine learning (ML) techniques are used to evaluate data, recognize patterns, learn from the recognized patterns, and make decisions. A ML system automatically and intelligently analyzes data to determine if a patient is worsening or improving. This faster method reduces hazards with early discovery and guarantees patient safety. Integrating ML with AAL technology over an IoMT framework would result in a better livelihood for citizens [5].

This work is primarily motivated by the constraints of the standard technique of diagnosis and the measurement of blood glucose in the human body, which traditionally places the glucose oxide sensor over the human body, which is intrusive. Furthermore, traditional models are only capable of forecasting problematic blood glucose levels. In a sensing activity, biosensors may monitor or sense the surroundings. A biosensor performs evaluations and records the results to transfer the information to a head office through a caregiver or linked localization. The base station receives the recorded data for further analysis. The primary goals of the current investigation are described in a point-by-point format below:

- We study similar technologies used in the continuous monitoring of patients and summarize the limitations to be addressed through our proposed model.
- We mechanize a model that would continuously monitor patients and sense their abnormal blood glucose levels in a non-invasive manner.
- We present a technique that would assist in converting HR signals to spectrogram images and label them accordingly for the training purpose.
- The features in the spectrogram images are localized, and any abnormalities in these spectrogram images are acknowledged.
- An alarm is generated, and a notification about the abnormal glucose level is sensed to provide better and timely treatment.
- The role of Ambient Assisted Living is demonstrated through an IoMT architecture over future perspective models.
- We analyze the proposed model’s results to assess the classification model’s performance.

The paper is structured as follows. Section 1 presents an introduction to the field of study, its motivation, and the study’s contributions. Section 2 offers literature on the existing models in assisted living. Section 3 presents the mechanism for converting signal data into spectrograms and labeling the images for training purposes. Section 4 presents the procedure for analyzing the spectrogram images to identify abnormalities. Section 5 presents associated hyperparameters, and Section 6 presents the results and discussion. Section 7 presents the IoMT integration in AAT and the future perspective model. Finally, Section 8 offers the conclusions of the study.

## 2. Background

Ambien-Assisted Living is a skilled nursing technology that utilizes ambient intelligence. It is possible to employ AAL to prevent, treat, and enhance the health and well-being of older persons. Elderly people may better manage their health issues with AAL technologies, such as through medication management systems and patient education [6]. Employing mobile emergency service technologies, accident detection technologies, and surveillance systems, AAL technology solutions may also enhance the safety of the elderly. The monitoring of daily activities (ADL) and the issue of alerts are further AAL solutions that aid in everyday activities. In addition, older individuals may use these technologies to better engage and interact with their peers and family members.

When it comes to modern communication, the IoT is a logical transition. The IoT allows many devices to interact, analyze, sense, and act. The elderly can live independently and safely with the help of IoT sensors mounted in AAL surroundings. To keep seniors as secure and autonomous as possible, many advanced technologies are utilized. Keep In Touch (KIT) [7] makes telemonitoring easier by using smart items and technology such as Near-Field Communication and Radio-Frequency Identification. Using KIT technologies, closed-loop medical services can analyze essential data and develop communication channels among elderly patients and their surroundings, as well as various groups of caretakers and medical practitioners. Closed-Loop Healthcare Services and KIT technology work together to create an IoT infrastructure in AAL situations. Age-related limitations, such as the inability to carry out essential daily tasks, the danger of falling, and chronic illnesses, including dementia, depression, and social isolation, may all be addressed using IoT solutions for AAL. Aging in one’s place of residence may be easier with IoT solutions and global services tailored to the requirements of the elderly, such as location-based services that promote functional independence.

**Figure 2:**
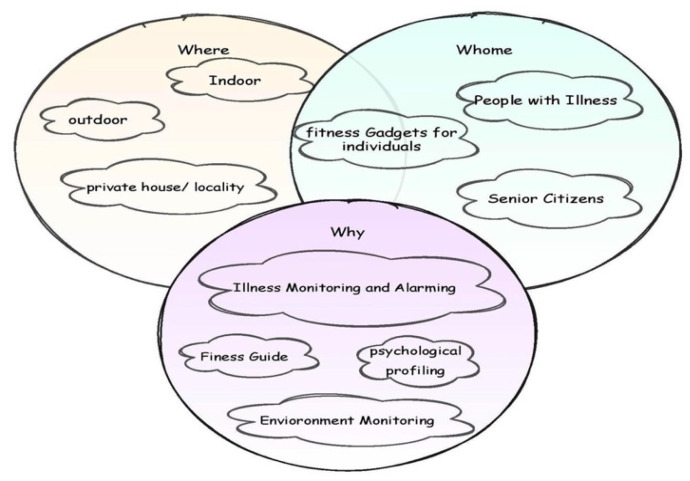
Ambient Assisted Living of healthcare management.

Developing an AAL system involves a broad range of considerations that may be stated in three words: who, where, and why, as shown in Figure 2. The technology has to keep track of individuals with various illnesses and senior citizens (whom). From a technical and operational standpoint, the deployment settings range from the indoors to outdoor situations (where). It is also important to note that an AAL system has various functions, from basic alarms to more comprehensive psychological profiling (why). In the current context, the proposed technology aligns with the healthcare conditions of senior citizens with diabetes in indoor and outdoor environments with the IoT framework through biosensors for the continuous monitoring and alarming of abnormal glucose levels.

Contextual factors, including private/public settings, confine the innovations that may be employed, whether they are intrusive or not, whether they function with longer or shorter range distance, etc. [8]. The characteristics that may be monitored can be drastically altered by using a variety of sensors. In addition, the kind of individuals to be watched, such as those with illnesses, those with impairments, and those who are otherwise healthy, significantly impacts the technology used. The technique of data-processing selection is undoubtedly an extra key feature of the development of AAL systems.

## 3. Literature Review

The human activity recognition model is a crucial aspect of AAL applications for promoting liberated living citizens and maintaining the standard of living for elderly individuals [9,10]. MapReduce technology is used over the cloud platform for training and detecting the activities of humans by processing mobile sensor data offloading to the cloud using K-nearest neighbor, naive Bayes, and iterative dichotomizer classifiers. These classification methods have been implemented using Hadoop and then evaluated through Elastic MapReduce. The authors used K-NN to categorize the mobile sensor data, which had a 71% accuracy [11]. The Home Event Recognition System (HOMER) [12] model allows for the integration and configuration of multiple home sensing devices and mobile robots, irrespective of underlying environmental setup, to offer specialized procedures such as sensing when an individual has fallen and has difficulty getting back up: an alarm can be activated.

The ActiveAdvice project [13], a European AAL-funded initiative conducted in six different countries, was created to fill these gaps by providing a web platform especially for senior citizens and their family members, AAL business representatives, and governmental organizations involved in aging issues throughout Europe. The architecture has offered a comprehensive market outline by displaying a catalog of AAL goods and services and advisory features that may enlighten and help people to discover a product that meets their requirements [14]. Pace et al. [15] presented INTER-Health, an interoperable IoT-driven service for delivering the ambient environment in diverse environments to identify abnormal circumstances. In recent years, various sensing devices have been downsized and made very energy-efficient. The most common wearable sensors include accelerometers, gyroscopes, and magnetometers, frequently placed on the patient’s hip or waist. These have been extensively employed for various purposes, including measuring a subject’s body posture, detecting and categorizing falls, and monitoring the gait cycle. Furthermore, these sensors are built into mobile devices such as smartphones and smartwatches, allowing for continuous monitoring of biological, behavioral, and environmental data [16].

Syed et al. [17] distinguished 12 physical activities of older persons with 97.1% accuracy. Wearable sensors would be placed over the subject’s left ankle, right forearm, and chest. Yassine et al. [18] offered a framework for IoT analytics data acquired from a smart home environment. The authors installed fog nodes between a home automation system and the cloud to direct processing resources from the cloud to the network’s edge. The cloud and fog computing systems perform activity identification, event detection, and psychosocial and predictive analytics. The outcomes are then sent to the home automation system for initiating the appropriate action. Jie et al. [19] have proposed a Time-Bounded Activity Recognition for Ambient Assisted Living over an IoMT framework. The Cumulatively Overlapping Windowing Approach for Ambient Recognition of Activities (COBRA) is used to recognize real-time scenarios, especially within 10, 30, 60, or 120 s after an activity’s start. COBRA employs novel sliding-window techniques and a logistic regression classification model to identify the task being performed.

A study named BrainSmart [20] presented the use of AAL technology over smartphone technology to recognize the activities and the falls of Parkinson’s disease patients. The technology uses accelerometers and gyroscope sensors to identify situations in the AAL environment. A study by Teller and Stivoric [21], carried out in 2004, has shown how technology may be placed into clothing or accessories such as bracelets and watches to assess, record, and relay various vital indicators. A study on situation awareness by AAL technology relies on machine-to-machine (M2M) technology [22,23]. It provides the adaptability needed by intelligent homes to help elderly citizens. The planned study will also have a substantial influence on patient monitoring, which is advanced by the smart city initiative. It is less usual to see socially assistive robots than wearable or ambient sensors, but this developing technology has the potential to improve human activities, particularly those involving physical exertion. In nursing homes, social-assistive robots are used for various tasks, such as transporting food or medicine carts, picking up objects for laundry collection, and more. They can also be used for transportation services, postal services, logistic support, trash logistics, and the logistics of cleaning materials. The quality of service and the happiness of residents and employees may rise due to the widespread use of these new technologies [24].

In addition to the technologies covered in this section, several other technologies may be used to provide healthcare services to consumers. There is a high need for wearable sensor technology that can monitor glucose levels in real-time for the elderly and warn their caregivers of their health. The suggested technology primarily focuses on providing timely medication for the patients, thereby enhancing the standard of livelihood of the elderly.

## 4. Data Acquisition and Labeling

This phase of the current research study focuses on acquiring the signal data from biosensors placed on the human body. The acquired data is then used in generating spectrogram images from the signal data and labeling the spectrogram images for further processing through deep-learning models. As a patient monitoring device that is non-invasive, these glucose instruments for data collection that give continuous blood glucose interpretations rely on the sensitivity and efficiency of biosensing devices to precisely and efficiently communicate blood glucose concentrations. Biosensors have three components: a bioreceptor, a signal transmitter, and an interface through which the signal can be interpreted [25]. Through the detection of analyte attachment to proteins or molecules, the device determines how much analyte has been deposited in a liquid sample. Binding and output options are diverse in biosensor categories. Affinity biosensors, which employ genes or antibodies to analyze the samples, are the second-most popular sensor. Sugar concentrations in many bodily fluids have been linked to blood glucose levels, including saliva, urine, tears, and interstitial fluid. In recent years, there have been positive achievements in the development of ubiquitous biosensing gadgets that measure blood sugar levels in these body fluids. Glucose deposits may be assessed using sweat, which is created by most people. A one-layer graphene-based biosensor with gold nanoparticles has been developed to detect glucose concentration in the blood via perspiration [26,27].

Various researchers have carried out a few similar studies in the recent past. A study on monitoring diabetic patients used the data classification technique with wi-fi (Ep8266) for older adults [28]. The other research direction deals with monitoring essential vital activities via smartphone technology using Near-Field Communication (NFC) activities [29]. Wang et al. [30] have discussed sensor models such as enzyme-free boronic acid-based glucose sensors and fluorescence-based glucose sensors that depend on blood glucose sensors for enhanced patient assessment and the management of blood sugar. Another case study-based evolution model assists chronically sick patients in accessing and using patient management services over the smartphone using the Transmission Control Protocol (TCP) and wi-fi technology for accessing the services [31]. In the current study, glucose oxide (GOD) sensors are used to analyze the body’s glucose levels in a non-invasive manner. Aspergillus niger is the source of glucose oxidase, which is stored as a solution. A plastic seal, anodic adhesive, and an ultrasonic connection are some of the existing micro-packing methods for packaging tiny amounts of glucose oxidase solution [32]. Low-temperature packaging is possible because parylene encapsulates glucose oxidase solution with UV adhesives. There are two parts to the package: a glucose oxidase solution-filled parylene capsule and an ultraviolet (UV)-curable adhesive cover for the capsule and reaction chambers. A rise in glucose concentration leads to higher output currents while the response time remains constant. The output of the GOD sensors is recorded in the range of −1.0–6.0 nA, depending on the blood glucose concentration [33].

**Assumption** **1:** *It is assumed that an output of the GOD sensor in the range of −1.0–2.0 nA is considered to be a low blood glucose level, 2.1–5.0 nA is assumed to be a normal blood glucose level, and observations above 5.0 nA are assumed to be high blood glucose levels.*

### 4.1. Spectrogram Generation

Many important signal components that are generally not recognized through detailed spectral analysis can be detected and identified by analyzing non-stationary components. This is especially true for components that only manifest themselves at a specific pace throughout device-operational transient states. The spectrogram images are transformed through Gabor transform. As a replacement for the Fourier transform, Dennis Gabor created the Gabor transform. There is a problem with Fourier transform, in that it only provides the signal’s frequency domain, but not its time domain. This is why a graph plotting frequency versus time may be created using the Gabor transform, a mix of the Fourier transform and the Gaussian distribution function. The Gabor transform’s Gaussian distribution function acts as a kernel, moving over the one-dimensional signals and calculating the Fourier transform and Gaussian function multiplication inside its frame to provide information on time when various frequencies occur [34,35]. The mathematical notation for the Gabor transform is represented in Equations (1)–(3):

(1) $$\hat{g}\left(s\right)=\overset{\infty }{\underset{-\infty }{\int }}g\left(x\right)e^{-isx}dx$$

(2) $$k_{a}\left(t\right)=e^{-{\left(t-\tau \right)}^{2/a^{2}}}$$

(3) $$T\left(g\right)\left(t,s\right)=\int_{-\infty }^{\infty }g\left(\tau \right)e^{-is\tau }k_{a}\left(\tau -t\right)d\tau$$

From the above equations, the variable $\hat{g}$ denotes the Fourier transform, and the Gaussian distribution function is denoted using the variable $k_{a}$. The variables t and s designate the time and the frequency, respectively. The symbol τ designates the frame’s center, and Gaussian functions represent spread.

### 4.2. Image Labeling

**Figure 3:**
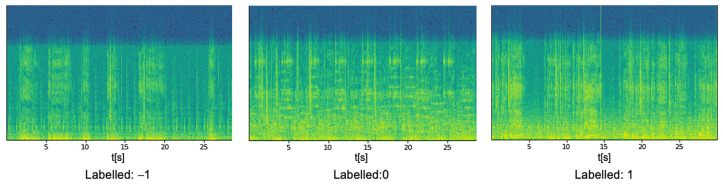
Spectrogram images represent the blood glucose levels.

In the spectrogram images, people with lower, normal, and high glucose levels are indicated as −1, 0, and 1, respectively. In the present study, the labeling of the spectrogram images is performed manually based on the real-time blood glucose levels of the patients. The labeled spectrograms are identified and used to train a model that can monitor them. For example, persons with low, high, or normal blood sugar levels are shown in spectrograms in Figure 3.

**Assumption** **2:** *Image labeling for training purposes is performed manually, which is tedious when labeling a large number of images. The images will be labeled automatically in real-time analysis for easy handling and precise outcome.*

### 4.3. Implementation Platform

The current study for alerting caretakers based on glucose intensities was simulated on an independent computer. The machine has an Intel^®^ Core i7 (11th Gen)—CPU@ 4.70 GHz and 16 GB of RAM running over a Windows 10 64-bit environment. The model runs on the Jupiter notebook V6.4.4 platform using the Anaconda package over libraries such as sklearn, PyTorch, NumPy, and pandas.

## 5. DenseNet-169 for Spectrogram Classification

The DenseNet-169 model is used in the process of classifying the spectrogram images that are obtained from the real-time surveillance of individuals. The spectrogram images are classified into low, normal, and high blood glucose levels. The model is trained with pre-existing data and utilizes input feature maps from previous sub-blocks to create a single input feature map for the current sub-block. A dense connection is essential to tackle vanishing gradient issues and minimize the number of parameters.

DenseNet-169 is a network that has 169 layers, and it is a prominent structure for various classification applications. It has significantly fewer trainable parameters compared to models with fewer layers. The DenseNet-169 network efficiently handles issues such as the vanishing gradient problem, has a strong feature distribution strategy, restricts the number of parameters, and encourages feature reuse, making it a dependable deep-learning architecture. Deep learning packages such as Tensorflow (Keras 2.8.0) and PyTorandes support DenseNet. Layers such as convolution, max_pool, dense (completely linked), and transition make up the architecture. The final layer of SoftMax is activated using ReLu throughout the model’s design. Using convolutional and max_pool layers, we may extract the image’s features and minimize the dimensions of our inputs. The flattened layer, which serves as an artificial neural system with a single array input, is followed by completely interconnected layers.

**Assumption** **3:** *Images are locally stored in the drive and used for training and testing the model. Generally, in AAL technology, the images are fetched directly from biosensors connected to the human body.*

**Figure 4:**
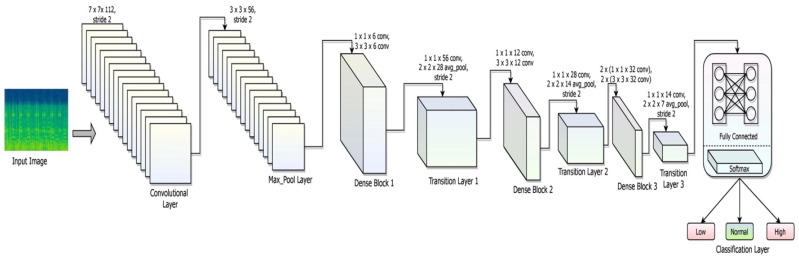
The layered framework of DenseNet-169 for classification.

The details of each network layer, such as the convolutional layer, the max_pool layer, the dense layer, the fully connected layer, and the soft_max layer of DenseNet-169, with detailed explanations of their responsibilities, are elaborated in this section. The layered framework of the DenseNet-169 is shown in Figure 4.

Convolution Layer: The activation of a convolutional layer is the consequence of applying a filter to an input. Repetition of filtering produces a feature map that shows the strength of each feature as it appears at various points in the input. Activation functions that are used in the current architecture are ReLU, which is applied to a feature map after it has been constructed using several filters. It is common to execute a dot product operation between a convolutional layer’s filter and the smallest input data. The square neuron component of size ℛ×ℛ is trailed by a convolutional layer of dimension p×p that results in a size output (ℛ−p+1)×(ℛ−p+1). This Equation shows how to combine the assistances from each of the primary layer cells to determine the non-linear feed to the component $s_{ij}^{l}$, shown in Equation (4) [36]:

(4) $$s_{ij}^{l}=\overset{p-1}{\underset{x=0}{\sum }}\overset{p-1}{\underset{y=0}{\sum }}\mu_{xy}{ℒ}_{\left(i+x\right)\left(j+y\right)}^{l-1}$$

The non-linearity of the model is assessed through Equation (5):

(5) $${ℒ}_{ij}^{l}=\lambda \left(s_{ij}^{l}\right)$$

Max_Pool Layer: Max pooling subsamples the tensors’ total dimension while keeping the depth of tensors constant. An overlapping max pool refers to adjoining windows for which the maximum value has been decided to overlap. The main advantage of including the max-pool layer would be producing a higher rate of convergence with a higher degree of generalization that is unaffected by scaling issues. It is linked to all or a subset of convolution layers. The max_pool of filter size k with the dimensions $\left(k_{x}\times k_{y}\times k_{z}\right)$ over the stride s assessed over the max_pooling layer $M_{p}$ is shown in Equation (6):

(6) $$M_{p}=\frac{\left(k_{x}-k+1\right)}{s}\times \frac{\left(k_{y}-k+1\right)}{s}\times k_{z}$$

Dense Layer: A dense layer is intimately coupled to its previous layer, which means that every neuron in the layer is connected to each neuron in the previous layer. A dense-layer neuron conducts matrix-vector multiplication over the input of each neuron in the previous layer. As indicated in the Equation, the usual formula for matrix-vector multiplication is as follows (7) [37]:

(7) $$X\cdot \eta =\begin{array}{ccccc} x_{11} & x_{12} & \ldots \ldots \ldots & x_{1n} & p_{1}\\ x_{21} & x_{22} & \ldots \ldots \ldots & x_{2n} & p_{2}\\ \vdots & \vdots & & \vdots & \vdots \\ \vdots & \vdots & & \vdots & \vdots \\ x_{m1} & x_{m2} & \ldots \ldots \ldots & x_{mn} & p_{m} \end{array}$$

The variable X in the above equation signifies the dimensions of vector m×n, and the variable p specifies another one-dimensional matrix 1×m. The symbol η designates the trainable parameters of the previous layer, updated via backpropagation in the training process. Backpropagation is used to change the weights associated with layer l denoted by the variable $l_{\omega }$ and the corresponding bias indicated by $l_{b}$, which are assessed by Equations (8) and (9) over the learning rate κ:

(8) $$l_{\omega }^{\prime }=l_{w}-\kappa \times dl_{\omega }$$

(9) $$l_{b}^{\prime }=l_{b}-\kappa \times dl_{b}$$

In the above equations, the variables $l_{\omega }^{\prime }$ and $l_{b}^{\prime }$ designate the new weight and the bias associated with layer l. The variables $dl_{\omega }$ and $dl_{b}$ denote the partial derivates of weight and bias of the loss function, assessed using the chain rule. The details of dense blocks are shown in Table 1.

Transition layer: The transition layer executes two activities: convolution and pooling, simultaneously, which are employed in a neural network model to minimize model complexity. A typical transition layer uses a 1 × 1 convolutional layer to minimize the sum of channels and a stride 2 filter to reduce the input dimensions by half. The transition layer performs downsampling operations. The details of the transition layer are shown in Table 2.

Global Average Pooling: As a substitute for the flattening layer following the final pooling layer of the convolutional neural network, global average pooling blocks, which do not have any trainable parameters, can be used. This basic technique greatly minimizes the input and qualifies the system for the subsequent classification layer. The global average pooling layer would assist in the removal of all trainable parameters and decrease the possibility of over-fitting, which must be addressed in fully linked layers through dropout. A tensor of dimensions l×b×h is shrunk to 1×1×d using global average pooling layers, which is a more extreme kind of dimensionality reduction. The mean of all lb values is all that global average pooling layers do to reduce each hb feature map to a single integer.

Fully Connected Layer: Each input neuron is linked to each output neuron in a neural network using a fully connected layer, which is a linear layer. Levels with complete connectivity classify data based on information gleaned from preceding layers. A fully connected layer has the significant benefit of being structure-agnostic, meaning that no particular assumptions about the input are required. A multilayer perceptron function (MPF) that attempts to translate the $x_{1}^{l}\times x_{2}^{l}\times x_{3}^{l}$ activation from the various previous layers is composed of a class probability distribution. Thus, the multilayer perceptron’s output layer will contain $x_{1}^{l-m}$ output neurons, where m designates the sum of layers using the MPF. The MPF is shown in Equation (10):

(10) $$p_{i}^{l^{\prime }}=f\left(\overset{x_{1}^{l}}{\underset{j=1}{\sum }}\omega_{i,j}^{l^{\prime }}\times p_{i}^{l}\right)$$

The goal of the entire fully connected structure would be adjusting the weight parameters $\omega_{i,j}^{l}$ to generate a probability interpretation of every category depending on the feature map created by the linear combination of the convolutional, non-linearity, rectification, and pooling layers.

Softmax Layer: The probability of input fitting to distinct classes would be evaluated by the softmax layer. The combination of all the possibilities is equal to one. Based on the likelihood of input data being correct, a classification is made. Low glucose, normal glucose, and high glucose are the three input categories in the proposed model. The formula for the softmax is shown in Equation (11).

(11) $$\sigma \vec{{\left(p\right)}_{i}}=\frac{e^{p_{i}}}{\sum_{j}^{k}e^{p_{j}}}$$

The input vector’s values are represented by the variable $e^{p_{i}}$ in Equation (11). Each member of the input vector is subjected to the conventional exponential function. This produces a positive number greater than zero, which is extremely low when the input is negative and high over the more significant input value. All values of the input vector are members of the softmax function’s input vector, and they may accept either any value that is positive, negative, or zero. Due to the normalization factor, it is possible to obtain a probability distribution from the softmax evaluation, where the denominator contains the normalizing factor. The softmax layer is associated with 3,029,214 parameters identified at the current layer through the activation function.

### Hyperparameters

**Figure 5:**
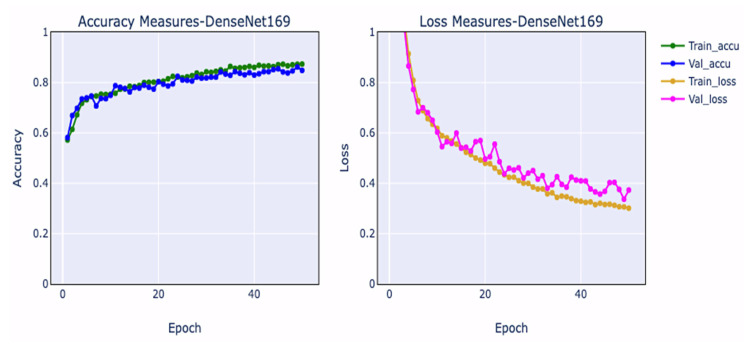
Graphs representing the hyperparameters of the model.

Training and validation loss measurements and training and validation accuracy metrics are crucial for evaluating the efficiency of the network and spotting overfitting contexts during network construction. Overfitting occurs when a model has learned too much from training samples, especially unpredictability, compromising its ability to assess the correct outcome from validation data. The training loss plot decreases as the number of epochs grows, but it does so before increasing. Underfitting occurs when a machine fails to learn from data and fails to generalize adequately across validation data. The training accuracy curve may be flat or have large loss values, suggesting that the model could not acquire knowledge from the training samples [38]. The performance curves associated with DenseNet-169 are shown in Figure 5.

**Figure 6:**
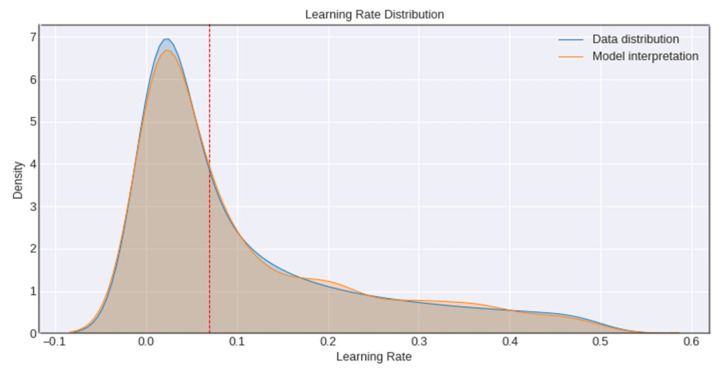
Graphs representing learning rate distribution.

The model’s learning rate is the other significant hyperparameter used in accessing the model’s performance. To begin with, the learning rate is raised linearly up to a certain value, and then it gradually decreases until it is zero. An overly high learning rate can lead to numerical instability because of the random distribution used to initialize the parameters of the model; however, training an initial model cautiously for a few epochs can allow us to use a higher learning rate later on in training, leading to a better classification performance. The network’s learning rate is drawn from a random distribution ranging various magnitudes in the expectation that most units will obtain a learning rate close to optimal, as shown in Figure 6.

## 6. Results and Discussion

**Figure 7:**
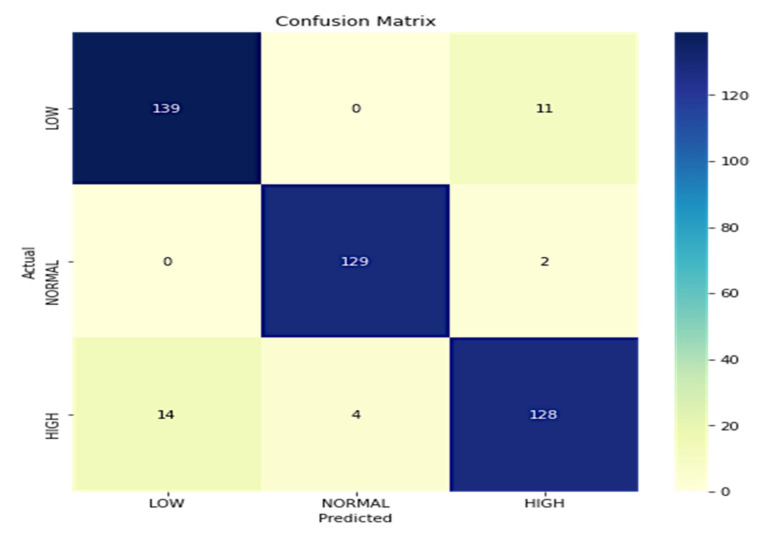
Confusion matrix of the DenseNet-169 model.

The model’s performance is measured following the ground facts linked with input images. The proposed model over DenseNet-169 is assessed by measuring the model’s true positive, true negative, false positive, and false negative instances. Based on the assessments, sensitivity, specificity, accuracy, and recall are calculated. A proper prediction of a normal glucose level is termed true positive, and a correct detection of an abnormal glucose level is considered to be true negative. Similarly, misinterpreting abnormal glucose levels as normal glucose levels results in a false positive, whereas misinterpreting normal glucose levels as abnormal glucose levels results in a false negative. In the current study, the images are classified into three classes, i.e., low, normal, and high glucose levels. Performance evaluations are also performed for each class. Figure 7 depicts the confusion matrix linked with the predictions. The precision and recall values of each class are independently shown in Table 3.

On further evaluation of the model’s performance, it is observed that it has exhibited an accuracy of 92.7% across all three classes. The efficiency of the DenseNet-169 model is evaluated vis-à-vis state-of-the-art models in a similar field by considering the metrics such as sensitivity, specificity, accuracy, and Matthews correlation coefficient (MCC), as shown in Table 4. There are only a few studies available that diagnose the real-time blood glucose level through spectrogram images. Electronic healthcare records (EHR) data deal with tabular datasets that include PIMA [39] and Luzhou [40] datasets.

**Figure 8:**
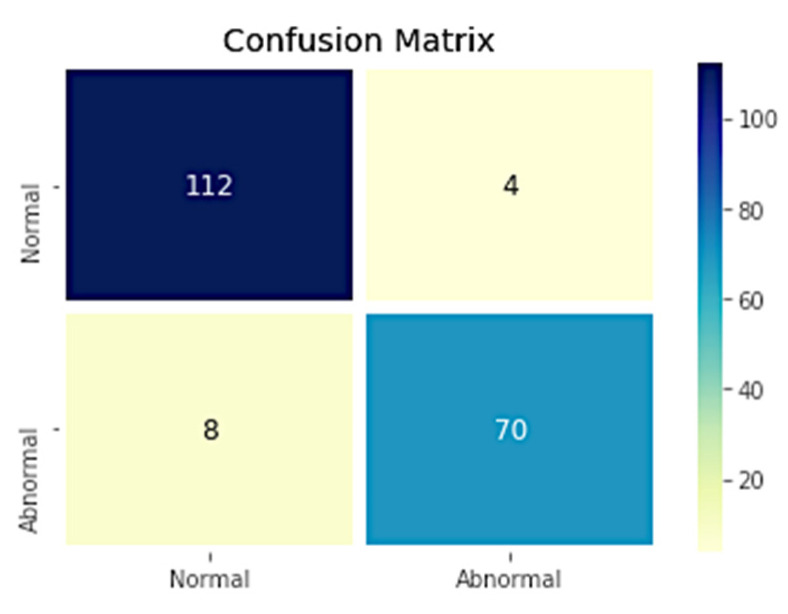
Confusion matrix for two classes.

The ablation study is carried over the same dataset by considering the two classes, i.e., the spectrogram images of normal glucose levels as one class and the samples with higher glucose levels as the other class, which are identified as the abnormal glucose level. The corresponding confusion matrix for the two classes is shown in Figure 8, and the evaluated metrics are shown in Table 5.

The ablation study has shown that the model’s performance depends on the number of classes. Table 5 shows that the model’s performance in the two classes is comparatively better than the three classes.

**Figure 9:**
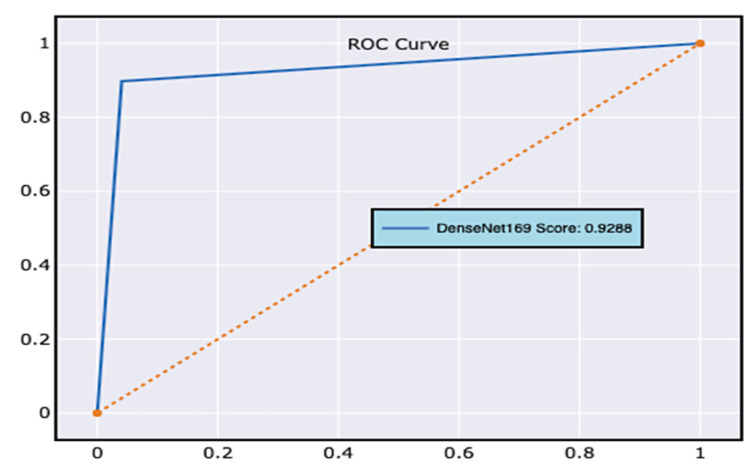
ROC curve of the DenseNet-169 model.

Other commonly used performance assessment indicators, such as the receiver operating characteristic curve (ROC curve) [42], assess the performance of the classification model. ROC curves consider both the true positive and the false positive measures. The ROC curve is generally applied to a model with binary classes. However, in the current study, the samples in the dataset are classified across three classes, i.e., low, normal, and high blood glucose levels. ROC curves are populated by considering the dataset as two samples, i.e., class 1, which holds all the samples of low and normal blood glucose levels, and class 2, which maintains the information of all the samples of higher blood glucose levels. The obtained ROC curve for the proposed model is presented in Figure 9.

**Figure 10:**
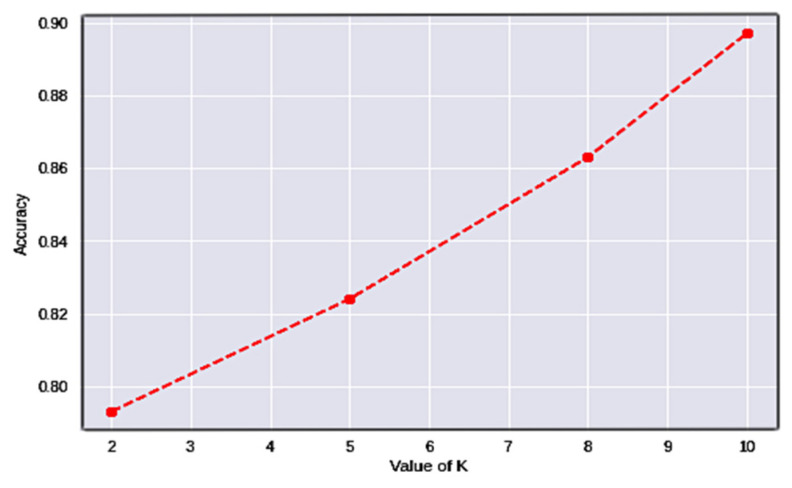
Cross-validation graph at various values of k.

Cross-validation (CV) [43] is a performance-assessment measure for classification issues that works by separating information into numerous folds and guaranteeing that each fold is used as testing data. The variable k designates the number of folds every testing data has to be divided into for validation. As a result, it is referred to as k-fold cross-validation. Table 6 shows the results attained after assessing the model through numerous folds, and Figure 10 shows the associated graphs.

Using k-fold cross-validation, the dataset is split into k non-overlapping folds. Each k-fold is given the option of operating as a test set, and the remaining folds are used together as a training dataset. The mean performance is presented after training and testing the k hold-out test sets. The values of k have been determined to be 2, 5, and 10, respectively. The results in Figure 9 demonstrate that the model consistently improves its performance with the intensification of the folds of data.

It can be observed from the tabulated values that the model’s accuracy increases with the increase in the number of folds. In the current study, the instances are categorized into three classes, considering the problem as two classes, i.e., high and low blood glucose levels, as the abnormal glucose levels would result in a binary class problem, whose accuracy is considerably better than a multi-class problem. Optimizing the network with weight optimization would improve the number of features considered in the evaluation process.

## 7. IoMT Integration in AAT

**Figure 11:**
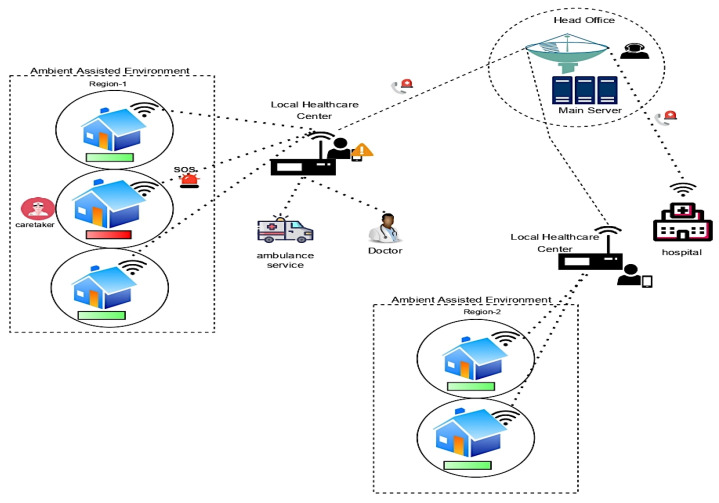
IoMT architecture diagram of future perspective technology.

The Internet of Medical Things (IoMT) is a subset of the Internet of Things (IoT) technology comprised of interconnected medical equipment for healthcare monitoring. IoMT devices, also known as healthcare IoT, integrate automating, interfacial sensors, and machine learning to provide human intervention-free healthcare monitoring [44]. IoMT technology links patients and physicians through medical devices, providing remote access to gather, analyze, and send medical data via a secure network. IoMT technologies help reduce needless hospital visits and related health expenses by enabling wireless surveillance of health indicators. Wearable, in-home, personalized, real-time health monitoring is covered under the IoMT medical technology category. The patient’s health condition in the Ambient assisted environment is consistently monitored, and abnormal situations alarm caretakers over the IoMT architecture. The IoMT architecture involves various networking nodes and sensor devices that work collaboratively. The integration of the nodes and devices is performed over an intelligent platform. The healthcare applications maintained by the caretakers would keep a complete log of the patient information and their health conditions. A local monitoring agent would notify a caretaker as to the abnormal situation that is prevalent for the client, i.e., the patient, and the head office would keep track of the patient’s condition and assist in reaching the associated hospital if the situation is out of control [45,46]. The local agents then generate an alert to the head office or the nodal station for their support. The head office would have direct access to hospital services. They might alert hospital staff based on the severity of the situation. The IoMT architecture diagram of future perspective technology is shown in Figure 11.

### Future Perspective Model

The user interface is exceptionally important for stakeholders’ easy access to technology. The real-time monitoring dashboard of the patient’s health situation could be accessible to the individual being monitored, his or her family members, the caretaker, and the agency that provides healthcare assistance. The interfaces for the individuals, family members, and caretakers are provided with a mobile application, and the healthcare agencies work on a desktop application in order to operate. The mobile system’s front-end interface might be utilized to install the suggested smart diagnostic technology for monitoring blood glucose levels. The model continuously acquires the data from the sensor devices over the IoMT architecture. The architecture’s back-end relies on services such as REST API and Flask, which connect the iOS platform to Kaggle. The model can be safeguarded with a user authentication technique and a secure socket layer (SSL). The user authentication at the caretaker’s end will ensure that access to the user data is provided only to a legitimate user, which functions by validating a username and the corresponding password of the user. SSL encrypts data as it is sent between a web browser and a server. SSL [47] encrypts the connection between a web server and a browser using the asymmetric key mechanism, ensuring that all data transmitted between parties remain private and secure. The user details and their monitored data are stored using NoSQL MongoDB. This provides people with a better feeling of authority over their sensitive data, i.e., the security and confidentiality of their health-related data.

**Figure 12:**
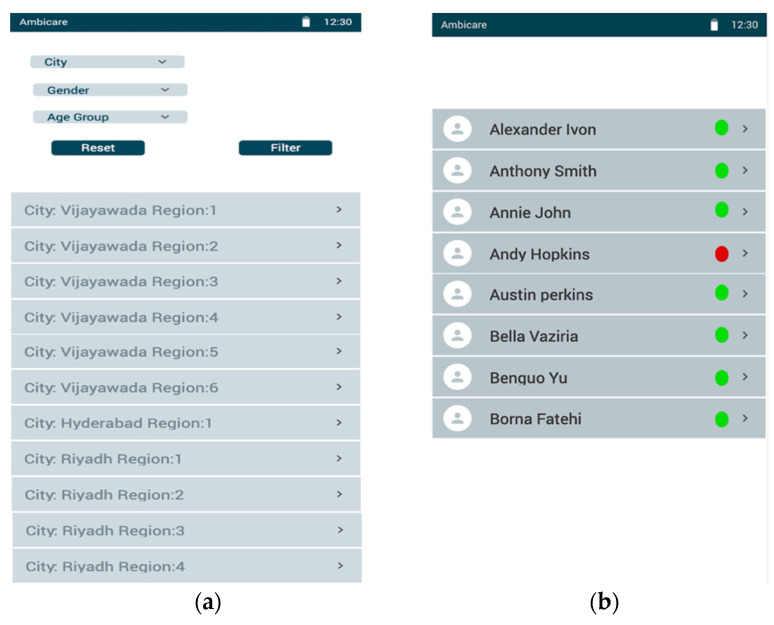
(**a**) The mobile screens for displaying the regions of AAL. (**b**) The list of associated users in each area and their active status.

**Figure 13:**
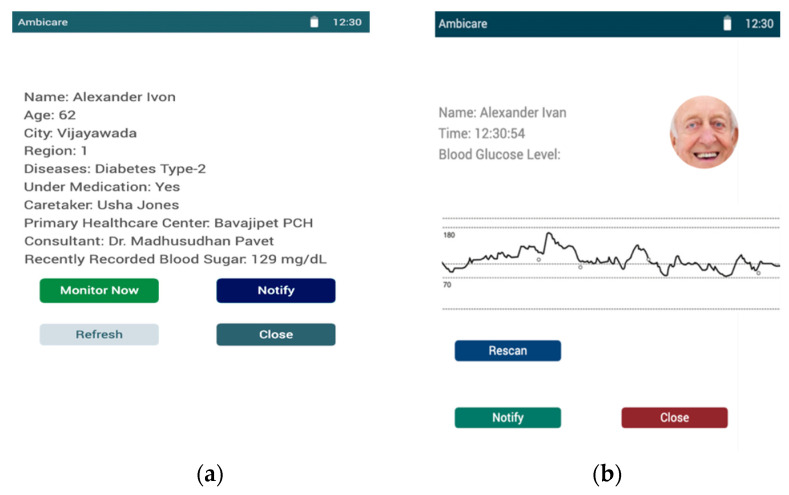
(**a**) The patient dashboard for displaying the patient details. (**b**) The screen for real-time monitoring of patients.

The technology outlined above can transform the future perspective model into a user-centric model with all the essentials. The mobile application’s user interface of the proposed model is shown in Figures. Figure 12 presents the dashboard of the caretaker, whereas Figure 12a shows a list of regions across the cities where Ambient assisted healthcare services are provided. Upon clicking on the respective regions in the city, Figure 12b shows the list of the patients whom the agency is monitoring. The details of each individual are provided to the caretakers of the associated region to monitor them remotely, as shown in Figure 13. Figure 12a presents the user dashboard in which details such as name, age, city, region, the disease being monitored, caretaker, nearby primary healthcare centers, consultant doctors, and recently recorded blood sugar levels are presented. Now, upon clicking on the monitor, the patient’s current blood glucose levels are presented, as shown in Figure 13b, in which a real-time graph is demonstrated with a provision to rescan to handle real-time errors.

Furthermore, as previously described in healthcare, real-time data analysis is a trend that is becoming a reality. Doctors may, in reality, check the historical healthcare data associated with patients. Doctors are also authorized to request information before drafting a report explaining the state of the patient’s health and prescribing the appropriate treatment. In such a context, the future perspective model would essentially assist in providing better patient treatment. Network latency and the privacy of the patient’s information are some of the potential challenges of the practical implication model [48]. The caretaker agent can access the patient data of all the users upon a successful login to the console. Therefore, it is favorable to regulate access to the limited records of the associated region to those whom the agent needs access. Latency is the other factor that determines any delay in response to the user query, which is usually incurred by processing in the business layer and networking delays. Such latencies are desired to be minimal, and future technologies must mechanize robust techniques that can efficiently handle the challenges mentioned above.

## 8. Conclusions and Future Scope

Many studies are impacting the outcomes and applications of assisted-living technology. They can monitor the overall health of people who live alone and may aid in reducing the overall expense of providing daily medical care for elderly citizens. The suggested system for continuously reviewing diabetic patients for better patient-centric treatment and lifestyle has shown fair effectiveness in recognizing clients with abnormal glucose levels and supporting caregivers in prompt treatment. The current study analyzes the spectrogram images generated from real-time HR signals using the DenseNet-169 model over three distinct classes. The performance is reasonably fair for the three-class problem, with an accuracy of 92.7%. The proposed model has exhibited a reasonable performance in monitoring the patients continuously in a non-invasive approach. Finally, the suggested technique is intended to supplement rather than replace current disease-diagnostic tools. When diagnosing a medical condition, laboratory results will almost always be more accurate than a remote examination alone.

The future perspective of the current study includes various divergent issues to be addressed, including the length of the generated spectrogram images. The current spectrogram images are of 25 s lengths. However, spectrogram images of 5 s and 10 s will not be the same. Further, the accuracies would differ, as the number of features is influenced by the duration of time. The security of the data exchanged among the sensor devices and base stations is the other major concern that must be addressed using robust encryption algorithms and maintaining the lifetime of sensor nodes using energy-aware algorithms to retain a better performance of sensing patients’ health conditions. The current work has been implemented over a limited dataset, and more samples are desired for optimal performance. In this context, deploying a self-learning model would result in a better performance. The technology could also be integrated with mobile application interfaces to make them easily accessible for the end users.

## Figures and Tables

**Table 1:** Details of the dense blocks used in the current study.

Layer	Kernel Size		
Dense Block 1	1 × 1 × 6 (Conv_operation)	Output: 56 × 56	Dropout factor 0.2
	3 × 3 × 6 (Conv_operation)		
Dense Block 2	1 × 1 × 12 (Conv_operation)	Output: 28 × 28	Dropout factor 0.2
	3 × 3 × 12 (Conv_operation)		
Dense Block 3	1 × 1 × 32 (Conv_operation)	Output: 14 × 14	Dropout factor 0.2
	3 × 3 × 32 (Conv_operation)		
Dense Block 4	1 × 1 × 32 (Conv_operation)	Output: 7 × 7	Dropout factor 0.2
	3 × 3 × 32 (Conv_operation)		

**Table 2:** Details of the transaction block used in the current study.

Layer	Kernel Size		
Transition Block 1	1 × 1 (Conv_operation)	56 × 56	Stride = 2
	2 × 2 (avgpool)	28 × 28	
Transition Block 2	1 × 1 (Conv_operation)	28 × 28	Stride = 2
	2 × 2 (avgpool)	14 × 14	
Transition Block 3	1 × 1 (Conv_operation)	14 × 14	Stride = 2
	2 × 2 (avgpool)	7 × 7	

**Table 3:** Precision and recall values of individual class.

	Overall True Samples	Precision	Recall
Class 1 (low)	153	92.6	90.85
Class 2 (Normal)	133	98.47	98.47
Class 3 (High)	141	87.67	87.67

**Table 4:** Performance analysis with the existing models for diabetes prediction.

Approach	Data Type	Sensitivity	Specificity	Accuracy	MCC
Decision Tree [41]	EHR data	0.781	0.561	0.744	0.762
Random Forest [41]	EHR data	0.789	0.661	0.840	0.436
Support Vector Machine [41]	EHR data	0.775	0.666	0.856	0.416
Naive Bayes [41]	EHR data	0.820	0.687	0.840	0.502
Finetuned AlexNet [27]	Spectrogram Images	0.826	0.725	0.925	0.484
Recurrent Neural Network	EHR data	0.837	0.774	0.818	0.591
Densenet-169	Spectrogram Images	0.912	0.881	0.927	~

**Table 5:** Ablation analysis against the number of classes.

Approach	Data Type	Sensitivity	Specificity	Accuracy
Densenet-169 (2-Classes)	Spectrogram Images	0.933	0.945	0.938
Densenet-169 (3-Classes)	Spectrogram Images	0.912	0.881	0.927

**Table 6:** Performance analysis with the existing models for diabetes prediction.

	K = 2	K = 5	K = 8	K = 10
DenseNet-169	0.793	0.825	0.862	0.897

## Data Availability

Not applicable for the current study.
